# The impact of environmentally-specific servant leadership on organizational green performance: The mediating role of green creativity

**DOI:** 10.3389/fpsyg.2022.1091025

**Published:** 2023-01-11

**Authors:** Hong Hou, Ruizhi Gai, Li An

**Affiliations:** ^1^College of Business Administration, Gachon University, Seongnam, Republic of Korea; ^2^College of Information and Control Engineering, Shandong Vocational University of Foreign Affairs, Weihai, China; ^3^School of Business Administration, Zhejiang Gongshang University, Hangzhou, China

**Keywords:** environmentally-specific servant leadership, green creativity, power distance orientation, organizational green performance, social learning theory

## Abstract

Due to the increasingly prominent environmental problems caused by excessive carbon dioxide emissions, many countries have put forward higher requirements for the green development of enterprises. Therefore, how to improve the green performance of enterprises has become a hot issue. Based on the social learning perspective, we examine the impact of environmentally-specific servant leadership on organizational green performance and test the mediating role of green creativity and the moderating role of power distance orientation. We test the theoretical framework by means of a questionnaire survey with a research sample of employees and their leaders working in the eastern coastal region of China. The results of the study suggest that: environmentally-specific servant leadership has a positive influence on organizational green performance; green creativity plays a mediating role between environmentally-specific servant leadership and organizational green performance; power distance orientation plays a moderating role between environmentally-specific servant leadership and green creativity; specifically, the higher the power distance orientation, the more significant the influence of environmentally-specific servant leadership on employees’ green creativity. This study enriches the research on environmentally-specific servant leadership and proposes a new perspective on how to improve green performance in organizations.

## 1. Introduction

With the acceleration of industrialization and urbanization, China has successively implemented national strategies such as “ecological industrial park,” “circular economy,” and “low-carbon city” pilot projects, and industrial policies such as “resource-saving and environment-friendly” enterprises, in order to promote the green transformation of social and economic development. As the concrete undertakers of social and economic development, enterprises are the key nodes of interaction between natural, economic, and social factors and the main body of developing green economy (Sun et al., 2022a). In other words, the implementation of enterprise green behavior becomes the micro foundation of the national macro green development strategy. In recent years, In recent years, environmental problems such as air pollution and global warming have become more and more serious, and more and more companies are incorporating green development into their core development strategies (Sun et al., 2022b). This is because business activities are not only the “perpetrators” of environmental issues, but also have a significant impact on the resolution. Gevrenova emphasized the important role of micro enterprises in the pursuit of environmentally friendly and green development (Gevrenova, 2015). Therefore, it is necessary to achieve environmental improvement by encouraging enterprises to pursue green development. Besides, with the gradual increase in consumer awareness of environmental protection, consumers are paying more and more attention to whether the products or services they buy are environmentally friendly, making green consumption more popular around the world. Consumer purchasing preferences will lead to changes in market trends. Concurrently, the green innovation capability and green image of enterprises become the core competitiveness of enterprises, and green performance also become an important indicator of healthy business development. Therefore, driven by the environmental pressure from the public and the competitive pressure from the market, developing green development strategies to enhance the green performance of enterprises has become the main development goal of each enterprise.

The success of a company in achieving the goal of pursuing green performance depends in part on its leaders. A growing body of research has recognized the importance of leadership on organizational green performance. In their study, Johnson and Schaltegger (2020) and Alwakid et al. (2021) pointed out that business leaders who aim to achieve both environmental and business goals have a transformative impact not only on their own sector, but also on the level of green development of the entire business. Environmentally-specific servant leadership has a management focus on caring for the environment and contributing to environmental protection, and therefore it is effective in enhancing organizational green performance. Some scholars have suggested to extend environmental servant leadership to the environmental context (i.e., concern for and service to the natural environment) to study its impact on corporate green development goals (Robertson and Barling, 2017). Established research generally agrees that environmentally servant leadership has a role in motivating employees to put effort into organizational green development or the pursuit of green performance (Chen et al., 2014). When leaders treat employees in an environmentally-serving way, employees are more likely to have access to the opportunities and resources they need to participate in the organization’s green activities, thus promoting the organization’s green performance (Dhar, 2016). For example, Tuan (2021) found that environmental servant leaders can improve the green performance of individual employees and teams by creating a green climate within the organization. And Luu (2020) found that environmentally-specific servant leadership are an effective way to improve organizational green performance by triggering more organizational environmental citizenship behaviors among employees. Although existing research provides preliminary insights into the role of environmentally-specific servant leadership in predicting organizational green performance, there is a lack of comprehensive understanding of the mechanisms of its effects on organizational green performance. Green creativity refers to the generation of original and useful ideas or solutions about green products, services, or practices (Dixon-Fowler et al., 2013). The green creativity of employees is the basis for the green development of an enterprise, especially the development and design of green products or services. At the same time, the green creativity of employees represents the overall competitive level of an enterprise, and is therefore of great significance to the green performance goals of an enterprise. Social learning theory proposes that individuals learn by observing and imitating the attitudes, values, and behaviors of role models. Leaders are the primary source of learning for employees in the workplace, especially when leaders are seen as role models (Wang et al., 2022). Therefore, employees will actively learn the leader’s values, attitudes, and knowledge about green development under the subtle influence and guidance of the leader (Dumont et al., 2017). Leaders who adopt an environmentally-specific servant leadership style focus more on training employees in green creativity and can give them more opportunities to participate in green development decisions. Therefore, environmentally-specific servant leadership helps employees to enhance their green creativity and further promote the improvement of organizational green performance. The social learning theory also states that the outcomes of social learning vary depending on the learner. Notably, heavily impacted by Confucian culture, Chinese people tend to have a higher power distance orientation than Westerners (Fu and Deshpande, 2014). The acceptance of the unequal allocation of power in institutions and organizations varies among people (Xia et al., 2022), despite the fact that China has a significant power distance. The leading role of leaders to employees is influenced by employees’ power distance orientation, and employees with high power distance orientation are more likely to respect and rely on leaders (Farooq et al., 2017), and therefore more likely to be influenced by leaders. Therefore, this study selected power distance orientation as the moderating variable to test the boundary role of employees’ power distance orientation between environmentally-specific servant leadership and employees’ green creativity.

The theoretical contributions of this manuscript are as follows: (1) Based on social learning theory, this manuscript explores the impact of environmentally-specific servant leadership on organizational green performance from a new perspective, which has enriched the research on environmentally-specific servant leadership to a certain extent. (2) By examining the mediating role of employees’ green creativity, the “black box” between environmentally-specific servant leadership and green performance is uncovered. (3) The existing literature barely pays attention to the boundary conditions of influencing factors of green creativity. In order to fill this gap, this manuscript explores the moderating effect of power distance orientation on environmentally-specific servant leadership and green creativity.

The rest of the manuscript is organized as follows: Section “2. Theoretical basis and research hypotheses” conducts the theoretical analysis and formulates the research hypotheses; Section “3. Research design” explains the variable measures and data sources; Section “4. Empirical analysis” presents the empirical results and discussion; Section “5. Conclusion and implications” draws conclusions, clarifies the theoretical and managerial implications, as well as points out research limitations and future research directions.

## 2. Theoretical basis and research hypotheses

### 2.1. Social learning theory

To investigate the relationship between environmentally-specific servant leadership and organizational green performance, we drew on social learning theory (Bandura, 1969), which suggests that individuals can acquire knowledge, skills, attitudes, and values by imitating influential role models (Bandura, 2001). Social learning theory states that although leaders often play the role of role models, not every leader can be seen as a role model for employees to learn from, i.e., there are certain limitations and constraints on whether subordinates choose to actively learn from the leader’s behavior. When leaders meet the conditions of being attractive, credible, and legitimate, subordinates are more motivated to imitate and learn from them.

In organizational contexts, social learning theory is often used to explain the behavioral transfer effect among organizational members (Bai et al., 2019), in which organizational members observe the behaviors of other members (including supervisors and colleagues) and then imitate those behaviors. Observational learning occurs especially between direct leaders and their subordinates, because supervisors are highly visible to their subordinates and there is frequent interaction and communication between subordinates and supervisors, which provides the basis for subordinates’ observational learning. Moreover, the superior leader has the power to reward and punish the subordinates, and the superior leader is perceived to be credible as an authority figure with abilities and traits that match his or her position (Brown et al., 2005). In addition, in the process of directing subordinates, most of the leader’s actions are directed to the subordinates, and thus subordinates are more likely to obtain information about organizational norms by observing the behavior of their immediate supervisors.

Environmentally-specific servant leadership promotes environmental values, attitudes, and actions in the workplace. Following the logic of social learning theory, by taking environmentally-specific servant leaders as role models, employees’ green creativity will be further stimulated, leading to improved organizational green performance (Afsar et al., 2018).

### 2.2. Environmentally-specific servant leadership

Servant leadership demonstrates a leader’s ethical responsibility to organizational growth, as well as to stakeholders, including employees and the community (Liden et al., 2014). Robertson and Barling (2017) suggest expanding the focus of servant leadership to environmental contexts and examine the role of environmentally-specific servant leadership in shaping environmental goals. Scholars have further expanded the focus of servant leadership to environmental sustainability and defined environmentally-specific servant leadership as role model leadership with pro-environmental values and to serve and help employees contribute to organizational and community sustainability. T,ăpurică and Ispăs,oiu (2013) defined environmentally-specific servant leadership as the ability of leaders to influence individuals and mobilize organizations to identify and achieve a corporate vision of sustainability, and the leadership that encourages positive environmental behavior in organizations. Environmentally-specific servant leadership encompasses the entire dynamic process by which individuals influence others to successfully implement environmental stewardship and environmental protection. Boiral et al. (2009) argued that environmentally-specific servant leadership can be viewed as charismatic leadership that motivates employees to perform their work tasks without damaging the environment based on a balanced economic and environmental relationship. Environmentally-specific servant leadership is a specific green leadership style under the broad structure of green leadership, which refers to the degree of willingness and commitment managers demonstrate to endorse green practices and change in order to gain a competitive advantage (Aboramadan et al., 2021). Servant leadership and transformational leadership share certain similarities but differ in their specific implementation (Graves et al., 2013; Eva et al., 2019). Specifically, green transformational leadership tends to achieve the organization’s sustainability goals and competitive positioning by motivating followers to contribute to the organization’s green goals (Jung et al., 2003; Schippers et al., 2008; Han et al., 2018), while environmentally-specific servant leadership tends to provide the necessary resources to encourage, serve, and help members become individuals with green values that contribute to the sustainability of the organization and the community.

### 2.3. Environmentally-specific servant leadership and organizational green performance

Organizational green performance refers to the performance of hardware and software involved in a company’s operations related to green products or processes, including energy saving, pollution prevention, waste recycling, green product design, or technological innovation in corporate environmental management. With the gradual increase in society’s demand for corporate environmental responsibility, green performance has become an important indicator of a company’s growth prospects (Feng and Wang, 2016). Companies are also recognizing the importance of green performance and the competitive advantage it brings (Robertson and Carleton, 2018), so many wise business leaders have considered green performance as a major corporate development goal.

The leader, as the “helmsman” of the company, plays a decisive role in the development direction and prospects of the company. Environmental service leaders are individuals with pro-environmental values and green goals, who have a specific preference for green performance and tend to focus on green development when formulating corporate development strategies (Hillebrandt and Barclay, 2022). As a source of green-related resources (e.g., knowledge, values, and support), environmentally-specific servant leadership leaders can help their employees build a platform of green-related resources. In an environment filled with green-related resources fostered by environmentally-specific servant leadership leaders, employees can develop and share positive perceptions of green values and norms (Hobfoll, 2001), and in turn, collaborate with employees in their efforts to green organizational performance. Therefore, we believe that environmentally-specific servant leaders have a positive influence on the green performance of companies. Specifically, environmentally specific servant leaders can encourage employees to contribute to organizational green performance by providing resources, empowering and guiding employees to become pro-environmental citizens (Popescu and Popescu, 2019). Social learning theory states that individuals will consciously imitate and learn from the behavior of important role models in the social system in order to acquire new behavior patterns. Environmentally servant leaders practice the green philosophy they promote and pursue the green growth of their companies. When leaders are seen as reliable role models, employees tend to learn the leader’s behavior and perform similarly. Therefore, with such leadership traits, environmentally-specific servant leaders can serve as providers and supporters of green-related resources for their teams and their team members in order to win the followers of their employees and thus unite all employees in their efforts to improve organizational green performance. In summary, this manuscript proposes the following hypotheses.

H1: Environmentally-specific servant leadership is positively related to organizational green performance.

### 2.4. The mediating role of employees’ green creativity

Creativity refers to the generation of novel ideas or solutions for a practice. Some scholars have extended creativity to the environmental field, proposing the concept of green creativity and defining it as the generation of original and useful ideas or solutions about green products, services, or practices. As the basis of general green innovation, green creativity plays a fundamental role in the development of green products.

Environmentally-specific servant leadership can provide employees with the necessary resources to motivate and help them engage in green practices, which is critical to the promotion of green creativity among employees. According to Ryan and Deci’s (2000) organismic motivational theory of self-determination, some social and environmental factors, such as leadership, can influence a person’s autonomous motivation. Leaders may encourage autonomous environmental drive in their workforce by helping them internalize green principles (Han et al., 2019; Li et al., 2022). Environmentally-specific servant leadership can stimulate the development of new ideas and encourage employees to be innovative in their work, so environmentally-specific servant leadership have a positive impact on organizational innovation. Environmentally-specific servant leadership encourage employees to engage in creative activities by enhancing their self-concept, task meaning, and environmental awareness (Kim et al., 2020). Furthermore, followers feel comfortable and supported to suggest and implement eco-initiatives in a green workplace atmosphere that environmentally-focused servant leaders foster. Employees are inspired to spend their resources in environmentally friendly activities and the creation of eco-initiatives above and above the basic requirements as more green resources (i.e., green-related knowledge and values) accumulate in such a supportive atmosphere (Luu, 2018; Tuan, 2020). Environmentally-specific servant leadership facilitate the development of new ideas in the innovation process and act as a catalyst by motivating followers to consider problems in new ways. According to social learning theory, when a leader is seen as a reliable role model, employees tend to follow the leader and learn from the attitudes, values, and behaviors the leader exhibits. Therefore, environmentally-specific servant leadership has a positive impact on employees’ green creativity when it provides support for employees and encourages followers to see problems from a new perspective. In turn, green creativity, as a fundamental driver of corporate green development, is important for companies to pursue green performance (Chen and Chang, 2013). Employees’ eco-initiatives are a contributing component to organizational green performance since individual creativity serves as a beginning point in innovation processes (Bidault and Castello, 2009). There is empirical support for the association between employee green creativity and the organizational green performance (Alt and Spitzeck, 2016). The green creativity of employees represents the overall green innovation level of an enterprise, which in turn involves the development and design of green products and services. When the overall level of green creativity of employees is high, it can ensure that the company can produce green products and services with good quality and low cost, which can enhance the competitiveness of the company and help the company pursue green performance. Therefore, this manuscript argues that environmentally-specific servant leadership helps to improve employees’ green creativity, and the improvement of employees’ green creativity provides the possibility for companies to pursue green performance. In summary, this manuscript proposes the following hypotheses.

H2: Employee green creativity plays a mediating role between environmentally-specific servant leadership and organizational green performance, that is, environmentally-specific servant leadership can improve organizational green performance by enhancing employee creativity.

### 2.5. Moderating effect of power distance orientation

Power distance orientation refers to the degree to which individuals accept unequal power distribution in institutions and organizations. The degree of acceptance of power inequality predicts how individuals interact with different levels of power. For example, employees with a higher power distance orientation are more aware of status differences during interactions, and they are more likely to defer to decisions made by superiors. Conversely, employees with lower power distance orientations are more concerned with equality with their leaders and less appreciative of them (Luo et al., 2020).

According to social learning theory, the influence of demonstrators on observers is influenced by observer differences. Consistent with this perspective, the present study hypothesizes that power distance orientation positively moderates the effect of environmentally-specific servant leadership on employees’ green creativity. As previously mentioned, individuals with higher power distance orientations are more likely to respect, obey, and trust authoritative leaders. Employees with high power distance orientation are more eager to carry out orders for environmental protection measures given by environmentally servant leaders, such as actively participating in training organized by the leaders. In addition, they are more likely to emulate the behaviors that environmentally servant leaders do to protect the environment and are more willing to apply the green knowledge they learn from their leaders at work to exercise and enhance green creativity than employees with a low power distance orientation (Van Dierendonck, 2011). Employees that have a higher power distance orientation are also more inclined to submit to authority, to respect and trust their leaders more, and to accept the demands of their superiors regarding green creativity. Therefore, employees with high power distance orientation are more likely to internalize the environmental friendliness of environmental servant leaders, prompting them to pay attention to environmental issues and enhance their own green creativity level. Employees with high power distance orientation show more respect and trust in authority, and they will learn the values held by environmental service leaders on their own. When environmental service leaders show values and attitudes related to protecting the environment in their work, people with high power distance orientation are more willing to emulate such values and show more environmental responsibility (Bhattacharya et al., 2022). Conversely, employees with low power distance orientation are more likely to view their leaders as equals and disobey the leader’s authority (Luo et al., 2020). For employees with a low power distance orientation, the leader’s values and friendly attitude toward the environment may not be worth learning. In addition, they are more likely to adhere to their own values than employees who see their leaders as role models. Therefore, employees with low power distance orientation do not readily endorse and accept orders given by their leaders and are reluctant to actively participate in training activities that help enhance their green creativity. In summary, this manuscript proposes the following hypothesis.

H3: Power distance orientation plays a moderating role between environmentally-specific servant leadership and employees’ green creativity. When employees’ power distance orientation is higher, environmentally-specific servant leadership has a stronger impact on the improvement of employees’ green creativity.

**FIGURE 1:**
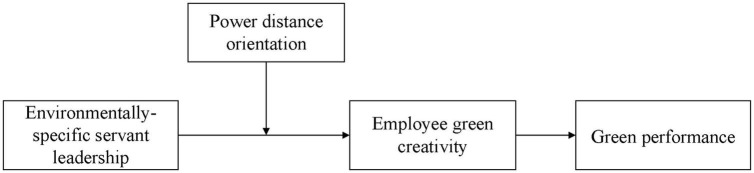
Theoretical model.

According to the research hypotheses, this study constructs a theoretical model as shown in Figure 1.

## 3. Research design

### 3.1. Sample selection and data collection

To test the research hypotheses of this manuscript, the research team collected primary data by distributing questionnaires to corporate employees and their direct leaders. From December 2021 to March 2022, the research team distributed 950 questionnaires to employees and leaders in the eastern coastal areas of China, such as Shandong, Jiangsu, Shanghai, Beijing, and Guangdong, through a combination of online and offline methods, and 705 questionnaires were effectively collected. Finally, the invalid questionnaires with a missing rate of more than 10% and those whose answers were obviously not in line with the requirements were checked one by one. A total of 576 valid questionnaires were obtained, and the effective recovery rate was 60.6%. The reason why the research team selected the above areas for data collection are as follows. Firstly, the eastern coastal region of China has a more active economy and a rich variety of industries, which can avoid the bias caused by a single industry to a certain extent, and the data analysis results are more generalized. Secondly, the eastern coastal region of China is often set up as a pilot region, and responds more actively to the requirements of national sustainable development and green development. At the same time, the communication with the international market is more frequent, so it pays more attention to environmental issues, which is more in line with our research needs. The specific sample distribution is shown in Table 1.

**TABLE 1:** Sample distribution statistics.

Demographic characteristics of the population		Number of people	Proportion
Gender	Male	324	56.25%
	Female	252	43.75%
Age	25–35 years old	181	31.4%
	36–45 years old	172	29.8%
	46–55 years old	153	26.6%
	55 years old and above	70	12.2%
Education level	High school or lower	57	9.9%
	College	143	24.8%
	Bachelor	296	51.4%
	Master	65	11.3%
	Doctor	15	2.6%
Type of industry	Manufacturing	58	10.1%
	Wholesale and retail	96	16.7%
	Trade	43	7.4%
	Internet industry	88	15.3%
	Restaurant and accommodation	158	27.4%
	Entertainment and culture	133	23.1%

*N* = 576.

### 3.2. Variable measurements

In this manuscript, all of the research variables were measured using internationally established scales, and in the process of developing the questionnaire, the research team asked English majors and scholars in related fields to work together to develop the questionnaire according to the standard translation-back translation procedure. After the double-blind translation, the back-translated scales were made sure that they were close to the original English version. Then, the questions were adjusted and modified according to the Chinese context. In the first pre-study, experts in related fields were invited to refine the questionnaire to ensure the appropriateness of the scale. In the second pre-study, MBA and EMBA students were invited to fill in the questionnaire, and the final questionnaire was further revised and improved according to the pre-study results. Except for the control variables, all other variables were measured on a 7-point Likert scale, with 1 to 7 indicating “completely disagree” to “completely agree,” depending on the respondent’s company situation. The relevant variables and specific items are described below: (1) the 12-item scale developed by Luu (2018) was chosen to assess environmentally-specific servant leadership, the sample items include “My leadership attaches importance to making contributions to environmental improvement,” “Business managers encourage environmental sustainability and the creation and maintenance of green values as a shared organizational vision,” “Enterprise leaders undertake the responsibility of environmental education and motivate organizations to carry out environmental management activities.” (2) The 6-item scale developed by Chen and Chang (2013) in their study was chosen to measure green creativity, with the sample items “the employee is good at finding creative solutions to environmental problems,” “I can propose new ways and methods for the organization to achieve environmental goals,” “I can propose new green concepts or ideas to improve the environmental performance of the organization.” (3) The 6-item scale developed by Luo et al. (2020) was chosen to estimate the power distance orientation, and the sample items are “My manager often makes the right decisions,” “Managers should make most decisions without consulting subordinates.” (4) The 4-item scale developed by Paillé et al. (2014) was used for green performance, sample items include “the firm’s products and services contain environmental values and sales are on the rise,” “Effectively reduce the emission or waste of harmful substances in the production and manufacturing process of enterprises.” (5) According to previous studies, demographic characteristics can have an impact on employees’ green creativity. In addition, the different types of industries will have a certain impact on the green performance of organizations. Therefore, this study selected four variables of gender, age, education level, and industry type to control, so as to reduce the influence of the above variables on the study results.

## 4. Empirical analysis

### 4.1. Common method bias and multicollinearity test

In this manuscript, the Harman one-way method was used to do the factor analysis of all entries in the validated questionnaire, and the results showed that the first factor explained 23.47% of the total variance without rotation, which did not exceed 50%. And the confirmatory factor analysis showed that the one-factor fit was poor (χ^2^/Df = 2.15, RMSEA = 0.138, RMR = 0.115, CFI = 0.701, TLI = 0.623). In addition, the variance inflation factor VIF values were all less than 2.5, and the tolerance between variables were all higher than 0.6. Therefore, there were no serious common method bias and multicollinearity problems in this manuscript, and the data were reliable.

### 4.2. Reliability and validity test

This manuscript used SPSS 26.0 and AMOS 24.0 software to conduct reliability and validity tests. The data results are shown in Table 2, and the Cronbach’α coefficient and the combined reliability (CR) of each variable are above 0.8, indicating that the variables have good internal consistency. In addition, according to the results of the confirmatory factor analysis in Table 3, the four-factor model have the best fit compared with other models (χ^2^/Df = 1.86, RMSEA = 0.023, RMR = 0.016, CFI = 0.943, TLI = 0.975), and the square root of AVE is greater than the correlation coefficient between any two variables (as shown in Table 4), indicating that the validity of the scale used in this study is better.

**TABLE 2:** Reliability and validity analysis.

Variables	Cronbach’α	CR	AVE
Environmentally-specific servant leadership	0.942	0.921	0.5640
Green creativity	0.864	0.878	0.5968
Power distance orientation	0.905	0.896	0.5880
Green performance	0.897	0.901	0.5864

**TABLE 3:** Confirmatory factor analysis.

	χ^2^	*Df*	RMSEA	RMR	CFI	TLI
Four-factor model (environmentally-specific servant leadership, green creativity, power distance orientation, green performance)	537.234	289	0.023	0.016	0.943	0.975
Three-factor model (environmentally-specific servant leadership—green creativity, power distance orientation, green performance)	747.923	293	0.077	0.066	0.881	0.868
Two-factor model (environmentally-specific servant leadership—green creativity—power distance orientation, green performance)	1093.666	296	0.101	0.083	0.791	0.771
One-factor model (environmentally-specific servant leadership—green creativity—power distance orientation—green performance)	1796.292	299	0.138	0.115	0.701	0.623

*N* = 576.

**TABLE 4:** Correlation analysis.

Variables	1	2	3	4	5	6	7	8
1. Gender	N/A							
2. Age	0.010	N/A						
3. Education	0.092	-0.003	N/A					
4. Type of industry	-0.010	-0.035	0.022	N/A				
5. Environmentally-specific servant leadership	-0.006	-0.002	0.057	0.049	0.751			
6. Green creativity	-0.002	-0.019	0.036	0.063	0.416**	0.773		
7. Power distance orientation	0.008	0.062	0.023	0.041	0.357**	0.369**	0.777	
8. Green performance	0.012	0.044	-0.049	0.086	0.360**	0.380**	0.381**	0.766
Mean	2.309	2.932	2.302	2.570	4.429	4.491	4.621	4.636
Standard deviation	0.780	0.730	0.913	0.842	0.760	0.800	0.888	0.849

*N* = 576; **p < 0.01. The numbers on the diagonal are the square root of AVE. N/A indicates not suitable for analysis.

### 4.3. Correlation analysis

The results of the correlation analysis between the variables are shown in Table 4. The correlations between environmentally-specific servant leadership, employees’ green creativity, power distance orientation, and green performance are all significant and the correlation coefficients are less than 0.5, which are within the acceptable range. The results of correlation analysis lay the foundation for hypothesis testing.

### 4.4. Hypotheses testing

To further verify the research hypotheses of this manuscript, the regression analysis of each variable was conducted by using SPSS26.0 software on the basis of correlation analysis, and the test procedure and results are shown in Table 5. Model 1 to Model 3 all have employee green creativity as the explanatory variable, and Model 4 to Model 7 all have organizational green performance as the explanatory variable. Model 1 and model 4 contain only control variables to test the effect of control variables on the explained variables.

**TABLE 5:** Regression results for directing, mediating, and moderating effects.

Explanatory variables↓	Green creativity			Green performance			
	Model 1	Model 2	Model 3	Model 4	Model 5	Model 6	Model 7
**Control variables**							
Gender	0.017	0.000	0.015	0.005	0.011	0.017	0.022
Age	0.047	0.036	0.023	0.064	0.069	0.047	0.052
Education level	-0.052	-0.084	-0.060	0.020	-0.100	-0.052	-0.073
Type of industry	0.089	0.065	0.073	0.042	0.007	0.089	0.062
**Independent variables**							
Environmentally-specific servant leadership		0.275*	0.17*		0.435**		0.175*
Mediator variable							
Green creativity						0.393**	0.278**
Moderator variable							
Power distance orientation			0.120*				
Interaction item							
Environmentally-specific servant leadership*power distance orientation			0.182**				
*R* ^2^	0.030	0.357	0.532	0.007	0.366	0.013	0.207
Δ*R*^2^	0.030	0.327	0.021	0.007	0.359	0.013	0.195
*F*	1.164	17.775**	28.023**	0.351	21.204**	0.659	9.592**
ΔF	1.164	113.933**	10.151**	0.351	72.847**	0.659	31.534**

*N* = 576; **p < 0.01, *p < 0.05.

#### 4.4.1. Directing effect test

Model 5 adds environmentally-specific servant leadership on the basis of Model 4, and the results show that there is a significant positive effect of environmentally-specific servant leadership on organizational green performance (*r* = 0.435, *p* < 0.05), and *R*^2^ increases by 0.359, lending support for hypothesis H1. This is consistent with the findings of Luu (2018), who noted that environmentally specific servant leadership has a significant positive impact on both team and individual green performance. Specifically, leaders have a decisive role in the development direction and prospect of the company. Environmentally-specific servant leaders are individuals with pro-environmental values who are keen on pursuing the green performance of the company (Luu, 2020), tend to reduce the impact of business activities on the natural environment in the course of business operations, and develop green development strategies to promote the improvement of the organization’s green performance. At the same time, environmentally-specific servant leaders are good at guiding and helping corporate employees to participate in green practices and encouraging them to contribute to the green performance of the company.

#### 4.4.2. Mediating effect test

This manuscript used the stepwise method to test the mediating effect of green creativity between environmentally-specific servant leadership and organizational green performance. As mentioned above, environmentally-specific servant leadership has a positive effect on organizational green performance, which satisfies the first condition for the existence of mediation. Model 2 adds environmentally-specific servant leadership to Model 1, and the results show that environmentally-specific servant leadership has a significant positive effect on employees’ green creativity (*r* = 0.275, *p* < 0.05), and *R*^2^ increases by 0.327, which satisfies the second condition for the existence of mediation. In Model 7, the regression coefficients of environmentally-specific servant leadership (*r* = 0.175, *p* < 0.1) and employee green creativity (*r* = 0.278, *p* < 0.05) are both significantly positive, and the effect of environmentally-specific servant leadership on organizational green performance decreased. According to the third condition for the existence of mediation, green creativity plays a partially mediating role between environmentally-specific servant leadership and organizational green performance. These findings provide further evidence for hypothesis H2. Combined with previous studies (Zheng et al., 2021), this manuscript suggests that the potential mechanism may be as follows. According to the social learning theory, the pro-environmental behaviors exhibited by the environmentally-specific servant leader at workplace and the company’s development strategies that are conducive to environmental protection will generate environmental intellectual stimulation and environmental vision motivation for employees. At the same time, leaders facilitate employees’ access to green resources at work, so that employees internalize the company’s green innovation goals as their own values and goals (Tuan, 2021) and promote their green creativity. With the cumulative effect, the improvement of employees’ overall green creativity will enhance the company’s ability to pursue green performance and thus increase the possibility of winning green performance.

#### 4.4.3. Moderating effect test

In this manuscript, after decentering environmentally-specific servant leadership and power distance orientation, a cascade regression was used to test the moderating effect of power distance orientation between environmentally-specific servant leadership and green creativity. Model 3 shows that the interaction term of environmentally-specific servant leadership and power distance orientation (*r* = 0.182, *p* < 0.05) has a significant positive effect on employees’ green creativity. Therefore, hypothesis H3 is verified. This finding validates previous literature that high power distance orientation enhances the impact of managerial leadership on employee behavior (Kwak and Shim, 2017). Xia et al. (2022) in their study also confirmed the moderating role of power distance orientation in the influence of environmentally-specific servant leadership on employees’ environmental self-accountability and low-carbon behavior. The potential mechanism may be as follows. Employees with high power distance orientation are more likely to obey their superiors’ leadership and more susceptible to their influence. When confronted with orders from environmentally-specific servant leaders for environmental protection measures, employees with high power distance orientation will respond more positively and take the initiative to enhance their own green creativity at the call of their leaders. In addition, compared to employees with low power distance orientation, they are more likely to imitate the behaviors that environmentally-specific servant leaders do to protect the environment and are more willing to apply the green knowledge they learn from their leaders in their work in order to exercise and enhance their green creativity.

## 5. Conclusion and implications

### 5.1. Conclusion

This study constructed a moderated mediation model based on social learning theory to explain the effects of environmentally-specific servant leadership on organizational green performance. Specifically, we examined the effect of environmentally-specific servant leadership leaders on organizational green performance through employee green creativity and tested the moderating role of power distance orientation between environmentally-specific servant leadership and green creativity. We used a questionnaire to collect data, and the results of the data analysis ultimately confirmed most of our initial hypotheses. The conclusions are as follows: (1) environmentally-specific servant leadership has a significant positive impact on organizational green performance. environmentally-specific servant leaders tend to reduce the impact of business activities on the natural environment during business operations and develop green development strategies to promote organizational green performance. (2) Employees’ green creativity plays a mediating role between environmentally-specific servant leadership and organizational green performance. That is, the contribution of environmentally-specific servant leadership to organizational green performance is mainly achieved by stimulating employees’ green creativity. (3) The power distance orientation plays a moderating role between environmentally-specific servant leadership and employees’ green creativity. The study in this manuscript yielded similar results to previous studies, employees with high power distance orientation are able to respond positively to the call of environmentally-specific servant leaders and actively participate in training activities to learn green knowledge and skills, and their green creativity improve faster.

### 5.2. Research implications

(1) This study focuses on the relationship between environmentally-specific servant leadership and corporate green performance, and has some theoretical contributions. This manuscript explores the role of environmentally-specific servant leadership in influencing organizational green performance from the perspective of social learning theory, and finds that environmentally-specific servant leaders are often regarded as reliable role models whose demonstrated attitudes and values toward the environment induce employees to follow and learn from them, thus contributing to the green development of the company. This manuscript clarifies the relationship between environmentally-specific servant leadership and corporate green performance from a new perspective and enriches the relevant literature. (2) By examining the mediating role of employees’ green creativity, this manuscript uncovers the “black box” between environmentally-specific servant leadership and green performance. Previous research has found that environmentally-specific servant leadership can create a green climate by promoting and practicing environmental values, and trigger employees’ environmental behaviors to improve corporate green performance. However, there is little literature examining how environmentally-specific servant leadership can contribute to the greening of their companies by enhancing the capabilities of their employees. This manuscript further confirms the indispensable role of employees’ green creativity in environmentally-specific servant leadership’ promotion of corporate green performance, thus providing a more comprehensive understanding of the mechanisms underlying the relationship between the two. (3) This study further explores the conditions under which environmentally-specific servant leadership has a stronger impact on employees’ green creativity. To fill this gap, this manuscript explores the moderating effect of power distance orientation on the relationship between environmentally-specific servant leadership and green creativity, as there is little focus on the boundary conditions of green creativity influencing factors in the existing literature. The results of this study show that employees with high power distance orientation are more motivated to learn from environmental servant leaders than those with low power distance orientation. Therefore, this finding provides a theoretical basis for enhancing the role of environmentally-specific servant leadership and how to improve employees’ green creativity.

### 5.3. Managerial implications

First, changing leadership style to more effectively pursue organizational green performance. Environmental pollution caused by the rough economic development method and the continuous increase of carbon emissions threatens the survival of human beings (Sun et al., 2023). As far as the natural environment is concerned, corporate business activities are one of the main causes of environmental pollution, and it is urgent for enterprises to change their development direction and actively assume environmental responsibility. Green performance is the main indicator to measure the degree of green development and environmental responsibility of enterprises. Therefore, business leaders should increase the importance of environmental issues, formulate corporate green development strategies, and actively pursue green performance. An effective starting point for companies is to change their leadership style and adopt environmentally-specific servant leadership to improve green performance. Second, striving to improve the green innovation ability of employees. The level of green creativity of employees represents the level of green innovation of a company, especially critical to the development and design of green products, and therefore determines the green competitiveness of a company and whether it can successfully win green performance. This manuscript helps to clarify the influence mechanism of environmentally-specific servant leadership on organizational green performance and draws leaders’ attention to improving employees’ green creativity. Environmentally-specific servant leaders should conduct relevant training activities so that employees can acquire relevant knowledge and skills and enhance their green creativity, thus contributing to the green performance of the company. Finally, making full use of the positive role of the power distance orientation in the relationship between Environmentally-specific servant leadership and employees’ green creativity. Business leaders should not only actively adopt Environmentally-specific servant leadership, but also set an example in their work so that they can.

### 5.4. Research limitations and future directions

(1) This study has certain limitations. Although the sample data collected in this manuscript is relatively adequate, it is limited to the eastern coastal provinces of China, and such data limit the judgment of the results. Future studies are encouraged to expand the scope of data collection, such as collecting data in different countries or different regions of China to obtain more generalized findings. (2) This manuscript only explored the role of environmentally-specific servant leadership in influencing employees’ green creativity, ignoring the influence of other organizational members on employees. Team members are more closely related to each other, and the words and actions of colleagues may have a more significant impact on employees’ green creativity and behavioral performance. We encourage future research to explore this relationship. (3) This manuscript only explored the moderating role of employees’ power distance orientation, however, employees’ own environmental awareness and environmental commitment can also influence their learning behaviors. We encourage future research to explore this relationship more fully.

## Data availability statement

The original contributions presented in this study are included in this article/supplementary material, further inquiries can be directed to the corresponding author.

## Author contributions

HH and RG predominantly contributed to conducting the literature review, designing the research, collecting some of the data, analyzing the data, and drafting the manuscript. LA repeatedly revised and refined the content of the manuscript. All authors substantially contributed to the research concept and design and approved the submitted version.
